# Dr. Johannes Muller

**Published:** 1858-07

**Authors:** 


JOHANNES MULLER, M.D., Professor of Physiology and Anatomy, died suddenly, while asleep, at Berlin, on the 28th of April, in the fifty-sixth year of his age. He was the author of an admirable work on "Physiology," which was translated into English by Dr. Baly, of London, and afterwards edited, with some modifications, by Dr. John Bell, of this city. He also produced a treatise on "Cancerous Formations," and contributed largely to German medical literature. Having always been regarded as authority on physiological subjects, the loss of such a man is truly to be lamented.

